# Sex-Based Analysis of Quality Indicators of End-of-Life Care in Gastrointestinal Malignancies

**DOI:** 10.3390/curroncol31030087

**Published:** 2024-02-22

**Authors:** Caitlin S. Lees, Hsien Seow, Kelvin K. W. Chan, Anastasia Gayowsky, Shaila J. Merchant, Aynharan Sinnarajah

**Affiliations:** 1 Division of Palliative Medicine, Dalhousie University, Halifax, NS B3H 2Y9, Canada; caitlins.lees@nshealth.ca; 2 Department of Oncology, McMaster University, Hamilton, ON L8V 5C2, Canada; seowh@mcmaster.ca; 3 Odette Cancer Centre, Sunnybrook Health Sciences Centre, University of Toronto, Toronto, ON M4N 3M5, Canada; kelvin.chan@sunnybrook.ca; 4 Institute for Clinical Evaluative Sciences (ICES), McMaster University, Hamilton, ON L8N 3Z5, Canada; anastasia.gayowsky@ices.on.ca; 5 Division of General Surgery and Surgical Oncology, Queen’s University, Kingston, ON K7L 2V7, Canada; shaila.merchant@kingstonhsc.ca; 6 Division of Palliative Medicine, Queen’s University, Kingston, ON K7L 3J7, Canada

**Keywords:** aggressive care, end of life, gastrointestinal cancer, hospital death, palliative care, quality indicators, sex

## Abstract

Indices of aggressive or supportive end-of-life (EOL) care are used to evaluate health services quality. Disparities according to sex were previously described, with studies showing that male sex is associated with aggressive EOL care. This is a secondary analysis of 69,983 patients who died of a GI malignancy in Ontario between 2006 and 2018. Quality indices from the last 14–30 days of life and aggregate measures for aggressive and supportive EOL care were derived from administrative data. Hospitalizations, emergency department use, intensive care unit admissions, and receipt of chemotherapy were considered indices of aggressive care, while physician house call and palliative home care were considered indices of supportive care. Overall, a smaller proportion of females experienced aggressive care at EOL (14.3% vs. 19.0%, standardized difference = 0.13, where ≥0.1 is a meaningful difference). Over time, rates of aggressive care were stable, while rates of supportive care increased for both sexes. Logistic regression showed that younger females (ages 18–39) had increased odds of experiencing aggressive EOL care (OR 1.71, 95% CI 1.30–2.25), but there was no such association for males. Quality of EOL care varies according to sex, with a smaller proportion of females experiencing aggressive EOL care.

## 1. Introduction

Sex and gender were found to significantly interact with social determinants of health, including socioeconomic status, education, geography, and employment. Ultimately, such factors underpin disparities in access to healthcare and the quality of the care provided [1,2,3,4]. Sex, which refers to biological, genetic, and physiologic characteristics distinguishing males and females, can be differentiated from ‘gender,’ which refers to socially constructed identities [5,6]. While it is clear that within intersectional frameworks, sex and gender impact health outcomes, there is a paucity of data regarding such relationships in the palliative care literature [6,7].

Quality indicators allow for an understanding of appropriateness, cost-effectiveness, and quality of patient care [8,9,10]. When considering health inequities, such quality indicators become particularly important to discern whether certain groups may be disadvantaged. The identification of inequalities can foster a thoughtful approach to identifying root causes, initiating change, and tracking change over time [4]. Within palliative care, in cases where patients are diagnosed with advanced and incurable cancer, quality indicators are used to measure whether patients receive supportive care for symptom management or potentially unwanted aggressive care in the last days of life [8,9]. Aggressive care, such as chemotherapy, emergency department visits, or admission to a hospital or an intensive care unit within the last weeks of life, is felt to be likely incongruent with clinical status and potentially indicative of inappropriate resource allocation or inadequate access to appropriate care planning and palliative supports [11]. Further, studies showed that such potentially inappropriate care at EOL is associated with the poorer well-being of relatives in the bereavement period [12]. Studies of the quality of end-of-life (EOL) care have previously demonstrated disparities between sexes and genders, with the male sex often being associated with the receipt of more aggressive care [11,13,14,15]; however, none of these studies directly compared rates of quality indicators by sex.

Our objective was to examine the impact of sex on the receipt of aggressive or supportive EOL care through a secondary analysis of previously published data in patients with gastrointestinal (GI) malignancies [16]. We found that overall, the quality of EOL care varies according to sex, with a smaller proportion of females experiencing aggressive EOL care.

## 2. Materials and Methods

This is a secondary analysis of data from a previously described population-based, retrospective cohort study of GI malignancy decedents in Ontario, Canada, from 1 January 2006 to 31 December 2018 [16].

### 2.1. Context

In the province of Ontario, healthcare is provided within a publicly funded framework in a variety of settings (community, acute care hospital, hospice, long-term care, etc.) and by a range of professionals (physicians, nurses, social workers, etc.) [17]. In the Canadian context, palliative care refers to the care provided to patients with a life-limiting illness, where the focus is on symptom management and end-of-life care [10]. Over the study period, there was increased investment in home care and palliative care services, palliative education for healthcare providers, and administrative infrastructure to support palliative care, as well as improvement in the palliative care billing framework and development of provincial performance indicators [18,19,20].

### 2.2. Data Sources

Administrative data from ICES (formerly known as the Institute for Clinical Evaluative Sciences) were used. Decedents were included if they had a diagnosis of a GI malignancy as per the *International Classification of Diseases, Tenth Edition* (ICD-10) codes in Table A1. Cancer subtypes were defined as malignancy of the anus, anal canal, or colorectal cancer; esophagus; gallbladder or biliary tract; liver; pancreas; small intestine; and stomach. GI malignancies comprise a significant portion of cancer deaths with a range of risk factors [21]. Decedents were excluded if death occurred within 30 days of diagnosis or if the diagnosis of cancer occurred on or after the date of death. Patients were also excluded if they were ≤18 years of age at death, did not have a valid provincial healthcare number, had no cancer diagnosis documented in the Ontario Cancer Registry, or did not have cancer documented as a cause of death.

### 2.3. Study Population

Decedents with a diagnosis of a GI malignancy were identified from the Ontario Cancer Registry (OCR), with data linkage to other ICES administrative datasets, including the Registered Persons Database (RPD), Postal Code Conversion File (PCCF), Canadian Institute for Health Information’s Discharge Abstract Database (DAD), National Ambulatory Care Reporting System (NACRS), Ontario Health Insurance Plan database (OHIP), Resident Assessment Instrument–Contact Assessment (RAICA), and Homecare Database (HCD). The data extracted from each dataset is specified in Table A2. Quality assurance and data stewardship are managed by ICES [22]. Variables included sex (which was then used to stratify the cohort), age at death, neighbourhood income quintile, rurality (defined as residence in a community with a population under 10,000), cancer type at diagnosis, and year of diagnosis. Survival was calculated from the date of diagnosis of the GI malignancy. Where the cause of death did not match the exact GI malignancy subtype, survival was calculated from the date of the most recent cancer diagnosis. Additionally, a modified Deyo–Charlson Comorbidity Index (DCCI) score was calculated based on ICD-10 codes from hospital admissions from between one and five years prior to death [23,24]. The GI malignancy resulting in death was excluded from this calculation.

### 2.4. Quality Indicators

Quality indicators of EOL care, previously defined by Henson et al. and used in the original analysis, were again employed [9]. Indicators included the following:

- ≥1 new hospitalizations in the last 30 days of life;
- ≥1 emergency department visits in the last 14 or 30 days of life;
- ≥1 new intensive care unit (ICU) admission in the last 30 days of life;
- Receipt of chemotherapy in the last 14 days of life;
- Physician house call in the last 14 days of life;
- Palliative home care nursing or support service in the last 30 days of life amongst those not hospitalized for the entirety of the period.

As in the previous analysis, aggregate indicators of aggressive and supportive EOL care were also created through the combination of individual indicators. Aggressive care was defined as experiencing one or more of the following in the last 30 days of life:

- ≥2 Emergency Department visits;
- ≥2 new hospital admissions;
- ≥1 new ICU admission.

Supportive care was defined as experiencing one or more of the following in the last 14–30 days of life:

- ≥1 physician house call in the last 14 days of life;
- ≥1 palliative care home care service in last 30 days of life (any provision of service recorded in the HCD and designated as ‘end-of-life’).

### 2.5. Statistical Analysis

Descriptive statistics were employed to delineate population characteristics at the time of death. Groups were compared using standardized difference (SD), where an SD ≥0.1 indicates a meaningful imbalance between groups [25]. The cohort was stratified by sex. The incidence and rate of each quality indicator were calculated for each sex and then further by year. The Cochran–Armitage trend test was used to investigate temporal trends in rates of quality indicators over time.

Multivariable logistic regression was used for each sex to calculate odds ratios (ORs) and 95% confidence intervals (CI) for factors associated with receipt of aggressive or supportive care. Covariates in the model included age at death, DCCI, survival, cancer type, income quintile, year of death (which was divided into three periods for ease of interpretation), and rurality. Covariates were chosen on the basis of previous studies and the authors’ clinical experience [16,26,27,28,29].

## 3. Results

In total, 29,529 females and 40,454 males were included in the study (Table 1). A breakdown of the study population by year is available in Table A3**.** A larger proportion of women were aged 80 years or older at the time of death [30]. Some differences were noted between sexes in income quintile, rurality, DCCI, and survival, though standardized differences were small. GI malignancy type varied according to sex, with a greater proportion of females diagnosed with colorectal, gallbladder, and pancreatic cancers, while a greater proportion of males were diagnosed with esophageal and liver cancer.

### 3.1. Quality Indicators

In comparing quality indicators (Table 2), a smaller percentage of females died in an acute care hospital bed compared to males (38.0% vs. 43.2%, SD = 0.11), though similar proportions of females and males received a palliative care service within the last year of life (93.3%, vs. 92.8%, SD = 0.02).

Of patients not hospitalized in the last 30 days of life, significantly fewer proportions of females had a new hospital admission in the last 30 days of life (47.2% vs. 54.4%, SD = 0.15), though there were no significant differences found between sexes with regards to new ICU admissions or palliative care homecare services.

Of patients not hospitalized in the last 14 days of life, again, a smaller proportion of females were found to have any ED visits (29.9% vs. 37.7%, SD = 0.17). However, there were no significant differences found between sexes in chemotherapy use or the receipt of a physician house call.

Quality indicator aggregates (Table 3) showed that, overall, a smaller proportion of females experienced aggressive care at EOL (14.3% vs. 19.0%, SD = 0.13), with a smaller proportion having at least two ED visits in the last 30 days of life (11.9% vs. 15.7%, SD = 0.11). Differences in hospitalizations and ICU admissions in the last 30 days of life were not significant. Despite the differences seen in the receipt of aggressive EOL care, there were no significant differences between sexes in receiving supportive care at EOL.

### 3.2. Trends over Time

**Figure 1:**
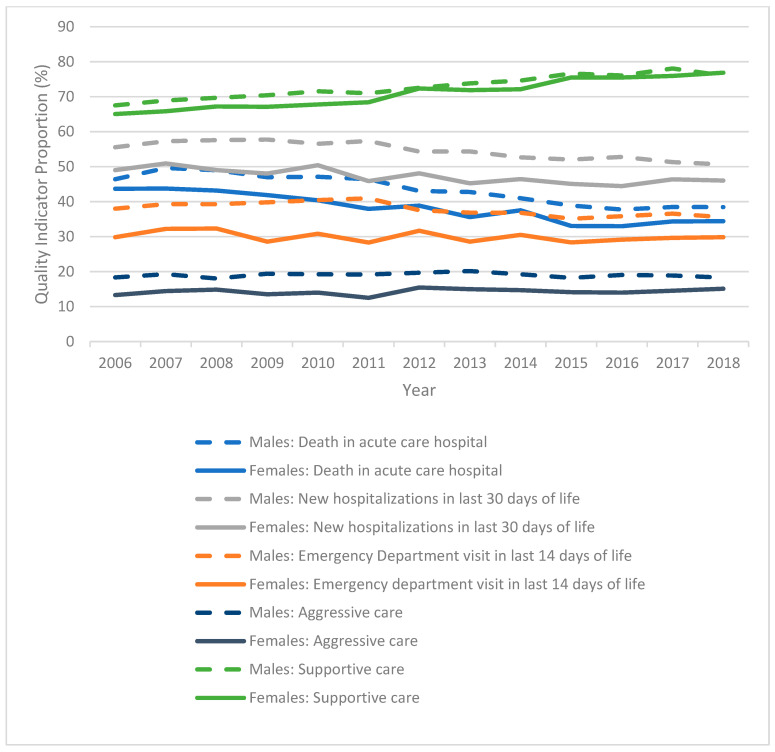
Quality indicator proportions by sex and year of death.

Trends for individual and aggregate indicators of aggressive and supportive EOL care are shown in Figure 1. For both sexes, rates of death in an acute care hospital bed (*p* < 0.001) and new hospitalizations (*p* < 0.001) in the last 30 days of life significantly decreased over time. For males, the rate of ED visits in the last 14 days of life decreased over time (*p* < 0.001), but there was no significant change seen for females (*p* = 0.072). There was no significant change in rates of aggressive EOL care for either sex (*p* = 0.186 for females, *p* = 0.833 for males), but rates of supportive EOL care increased for both sexes over the study period (*p* < 0.001).

### 3.3. Factors Associated with Aggressive and Supportive Care

Results of the multivariable logistic regression models of aggregate quality indicators, both aggressive and supportive care, are shown by sex in Table 4 and Table 5, respectively. The odds of receiving either aggressive care or supportive care varied between sexes according to age at death, survival, cancer type, and income quintile.

Females aged 18–39 had significantly increased odds of aggressive EOL care, while there was no age bracket for males with such an association. For both sexes, being over the age of 60 was associated with reduced odds of aggressive EOL care. The comorbidity index was found not to be associated with aggressive care for either sex; however, females with a comorbidity score of 1 or more had significantly reduced odds of supportive EOL care.

There was considerable variability by cancer type in association with odds of aggressive or supportive EOL care. For females, all cancer types were associated with increased odds of aggressive EOL care when compared to anal and colorectal cancers, while for men, such an association was only found for esophageal, liver, and gastric cancers. For both males and females, gallbladder/biliary cancers, pancreatic cancers, and gastric cancers were associated with increased odds of supportive EOL care.

The income quintile was not significantly associated with the odds of receiving aggressive care for females, but there was an association between lower income quintiles (quintiles 1–3) and aggressive care for males. Both males and females in the lowest two income quintiles had reduced odds of experiencing supportive EOL care. Residency in a rural area was found to be associated with increased odds of supportive EOL care and aggressive EOL care for both sexes. A later year of death within the study period was found to be associated with increased odds of receiving supportive care at the end of life, though there was no such association between receipt of aggressive care and year of death.

## 4. Discussion

This secondary analysis elaborates on our previous work and highlights sex-based differences in aggressive and supportive EOL care in patients with GI malignancies. Generally, rates of aggressive care remained stable over time, while rates of supportive care increased for both sexes. However, significantly more males experienced aggressive EOL care when compared to females. Further, our previous work found that younger age, residency in the three lowest-income quintile neighbourhoods, and rurality were associated with increased odds of experiencing aggressive care at EOL. However, sex was not included as a predictor in this model [16]. In this secondary analysis, we found that the female population may be driving the associations between increased odds of aggressive care at EOL and younger age. Conversely, the male population may strongly contribute to the association between aggressive care EOL and lower income quintiles.

More generally, females experienced less aggressive care at EOL, when compared to males. This greater resource intensity of EOL care for males is generally consistent with other studies that showed males are more likely to experience aggressive care at EOL [11,13,15,29]. The lack of significant difference between sexes in the use of supportive EOL care is somewhat surprising, given a number of studies showing that females are more likely to prefer palliative care in the context of serious illness [13,31,32]. Other studies found that females are more likely to understand that their disease is incurable [33] and be aware of palliative care [34]. Such preferences and insight into prognosis would typically be necessary to arrange home care or a physician house call, which were used as indices of supportive EOL care in our study.

We found an association between younger age and increased odds of aggressive EOL care in females, yet not males. Other social factors not captured in our data may be driving this association. Previous research found reduced odds of aggressive care at EOL in older age cohorts regardless of sex [11,14,15,35]. One study of patients with hematologic and solid tumour malignancies found that female sex and age ≤45 years were associated with receipt of chemotherapy in the last 14 days of life, another indicator of aggressive EOL care [36]. Similarly, a study of lung cancer decedents found a stronger association between aggressive care at EOL and females under age 50, as compared to males [37]. While the multivariable logistic regression model accounted for cancer type, comorbidities, rurality, and income, we did not capture other relevant social variables, such as family status, children, and traditional gender roles, that may influence treatment received. Previous studies showed that family status and gender roles do influence preferences for treatment in patients with cancer [38,39,40].

We also found an association between aggressive care at EOL and lower income quintile for males, yet not females, though the differences were only marginally different, making the true significance difficult to interpret. A study of US patients found that while income was not associated with treatment preferences, lower education level was noted to be associated with a desire for more aggressive care [41]. It is possible that a lower income quintile is a surrogate marker of education level for males. The same relationship, however, may not appear for females, who, despite education level, may have lower incomes due to the longstanding gender wage gap in addition to traditional gender norms driving increased caregiver responsibilities and less income-generating work outside the home [42,43]. Other studies showed an association between increased risk of aggressive EOL care and lower income [16,44,45,46], though, to our knowledge, this is the first study to directly compare how income may affect the aggressiveness of care for each sex.

As with our initial analysis, this study is limited by the retrospective and observational nature of our study design. The use of data from a single province may limit generalizability. The use of administrative data also creates some limitations in collecting other relevant data on gender identity, intersex individuals, and how sex and gender intersect with other social determinants. Factors impacting end-of-life decision-making, such as patient preferences, family preferences, and doctor–patient communication, are also missing, nor did this study investigate congruence between advance care planning and the end-of-life care received. Lastly, our definitions of aggressive and supportive care do not capture the entire realm of elements within aggressive and supportive care, so we are only able to provide a partial picture.

Future studies would benefit from the collection of a full spectrum of social determinants of health, gender identity, and sex. An analysis of the relationship between supportive and aggressive care interventions would allow us to better examine how supportive care interventions may decrease potentially inappropriate, aggressive EOL care, particularly within populations facing health inequities.

## 5. Conclusions

This secondary analysis of quality indicators of EOL care over 13 years in a Canadian province provides novel and useful information regarding how sex may influence the quality of care for patients. While rates of supportive care at EOL are increasing over time for both sexes, rates of aggressive care remain stable. Significantly fewer females experience aggressive care at EOL, suggesting that females may receive better quality care at EOL as compared to males.

## Figures and Tables

**Table 1:** Characteristics of the study population.

Descriptive Statistics		Female		Male		Standardized Difference
		N = 29,529		N = 40,454		
		N	%	N	%	
Age at death	18–39	316	1.1%	346	0.9%	0.02
	40–49	1122	3.8%	1555	3.8%	0.002
	50–59	3170	10.7%	5665	14.0%	0.10
	60–69	5862	19.9%	10,268	25.4%	0.13
	70–79	8106	27.5%	12,088	29.9%	0.05
	80+	10,953	37.1%	10,532	26.0%	0.24
Income quintile	1 (lowest)	6420	21.7%	8349	20.6%	0.03
	2	6333	21.4%	8723	21.6%	0.003
	3	5832	19.8%	7967	19.7%	0.001
	4	5464	18.5%	7651	18.9%	0.01
	5 (highest)	5403	18.3%	7619	18.8%	0.01
	Missing	77	0.3%	145	0.4%	0.02
Rural	Urban	25,888	87.7%	34,858	86.2%	0.05
	Rural	3620	12.3%	5550	13.7%	0.04
	Missing	21	0.1%	46	0.1%	0.01
Comorbidity index	0 or missing	16,640	56.4%	21,631	53.5%	0.06
	1+	12,889	43.6%	18,823	46.5%	0.06
Survival	Mean ± SD (months)	2.24	4.19	2.09	3.44	0.04
	1–3 months	6140	20.8%	7930	19.6%	0.03
	3–12 months	9921	33.6%	13,326	32.9%	0.01
	1–5 years	10,276	34.8%	14,932	36.9%	0.04
	5+ years	3192	10.8%	4266	10.5%	0.01
Cancer subtype	Anal/Colorectal	12,453	42.2%	14,502	35.8%	0.13
	Esophagus	1958	6.6%	6342	15.7%	0.29
	Gallbladder/Biliary tract	2950	10.0%	2733	6.8%	0.12
	Liver cancers	918	3.1%	3190	7.9%	0.21
	Pancreas	7790	26.4%	8174	20.2%	0.15
	Small intestine	442	1.5%	507	1.3%	0.02
	Stomach	3018	10.2%	5006	12.4%	0.07

**Table 2:** Quality indicator rates by sex.

Indicator	Female		Male		Standardized Difference
	N	%	N	%	
**Full Study Population**	**29,529**		**40,454**		
Death in acute care hospital bed	11,228	38.0%	17,493	43.2%	0.11
Any palliative care service in the last year of life	27,541	93.3%	37,535	92.8%	0.02
**Patients not hospitalized during last 30 days of life**	**27,244**		**37,536**		
New hospitalization in last 30 days of life	12,850	47.2%	20,433	54.4%	0.15
New ICU admission in last 30 days of life	1233	4.5%	2275	6.1%	0.07
Any palliative care homecare service in last 30 days of life	18,656	68.5%	26,671	71.1%	0.06
**Patients not hospitalized during last 14 days of life**	**24,510**		**33,596**		
Emergency department visit in last 14 days of life	7334	29.9%	12,669	37.7%	0.17
Chemotherapy use in last 14 days of life	741	3.0%	1417	4.2%	0.06
Physician house call in last 14 days of life	7672	31.3%	10,248	30.5%	0.02

**Table 3:** Quality indicator aggregates by sex.

Indicator	Female		Male		Standardized Difference
	N	%	N	%	
**Patients not hospitalized during last 30 days of life**	**27,244**		**37,536**		
**Aggressive care**	**3893**	**14.3%**	**7128**	**19.0%**	**0.13**
At least 2 ED visits in last 30 days of life	3236	11.9%	5901	15.7%	0.11
At least 2 new hospitalizations within last 30 days of life	1769	6.5%	3346	8.9%	0.09
New ICU admission in last 30 days of life	1233	4.5%	2275	6.1%	0.07
**Supportive care**	**19,406**	**71.2%**	**27,447**	**73.1%**	**0.04**
Physician house call in last 14 days of life	7695	28.2%	10,276	27.4%	0.02
Any palliative care homecare service in last 30 days of life	18,656	68.5%	26,671	71.1%	0.06

**Table 4:** Multivariable logistic regression models for supportive care by sex.

Effect	Level	Female			Male		
		OR	Lower	Upper	OR	Lower	Upper
Age at death	18–39	1.13	0.82	1.57	1.10	0.82	1.48
	40–49	**1.30**	**1.07**	**1.58**	**1.18**	**1.02**	**1.38**
	60–69	**0.87**	**0.77**	**0.97**	**0.87**	**0.80**	**0.94**
	70–79	**0.67**	**0.61**	**0.75**	**0.73**	**0.68**	**0.79**
	80+	**0.43**	**0.39**	**0.48**	**0.52**	**0.48**	**0.56**
	50–59 (REF)	1.00			1.00		
Deyo–Charlson comorbidity index	1+	**0.88**	**0.83**	**0.93**	0.96	0.92	1.01
	0 or missing (REF)	1.00			1.00		
Survival	3–12 months	**1.54**	**1.43**	**1.66**	**1.63**	**1.52**	**1.74**
	1–5 years	**1.65**	**1.53**	**1.78**	**1.68**	**1.57**	**1.79**
	5+ years	1.06	0.96	1.17	**1.09**	**1.00**	**1.19**
	1–3 months (REF)	1.00			1.00		
Cancer subtype	Esophagus	1.07	0.96	1.20	**1.13**	**1.05**	**1.22**
	Gallbladder/Biliary tract	**1.21**	**1.10**	**1.34**	**1.13**	**1.03**	**1.25**
	Liver cancers	1.01	0.87	1.18	**0.84**	**0.77**	**0.91**
	Pancreas	**1.47**	**1.37**	**1.58**	**1.43**	**1.33**	**1.53**
	Small intestine	1.05	0.84	1.31	1.12	0.90	1.39
	Stomach	**1.13**	**1.03**	**1.25**	**1.10**	**1.01**	**1.19**
	Anal and colorectal (REF)	1.00			1.00		
Income quintile	1 (lowest)	**0.69**	**0.63**	**0.75**	**0.70**	**0.64**	**0.74**
	2	**0.83**	**0.76**	**0.90**	**0.85**	**0.79**	**0.92**
	3	**0.90**	**0.83**	**0.99**	0.92	0.86	1.00
	4	0.96	0.88	1.06	**0.92**	**0.85**	**0.99**
	5 (highest—REF)	1.00			1.00		
Rural status	Rural	**1.19**	**1.09**	**1.30**	**1.25**	**1.17**	**1.34**
	Urban (REF)	1.00			1.00		
Year of death	2011–2014	**1.23**	**1.15**	**1.31**	**1.19**	**1.12**	**1.26**
	2015–2018	**1.58**	**1.48**	**1.69**	**1.47**	**1.39**	**1.56**
	2006–2010 (REF)	1.00			1.00		

Note significant results are bolded.

**Table 5:** Multivariable logistic regression models for aggressive care by sex.

Effect	Level	Female			Male		
		OR	Lower	Upper	OR	Lower	Upper
Age at death	18–39	**1.71**	**1.30**	**2.25**	1.05	0.80	1.38
	40–49	1.04	0.86	1.24	0.98	0.85	1.12
	60–69	**0.86**	**0.76**	**0.96**	**0.83**	**0.76**	**0.90**
	70–79	**0.65**	**0.58**	**0.73**	**0.76**	**0.70**	**0.82**
	80+	**0.42**	**0.38**	**0.47**	**0.53**	**0.49**	**0.58**
	50–59 (REF)	1.00			1.00		
Deyo–Charlson comorbidity index	1+	1.01	0.94	1.08	0.98	0.92	1.03
	0 or missing (REF)	1.00			1.00		
Survival	3–12 months	**0.82**	**0.75**	**0.90**	**0.83**	**0.77**	**0.89**
	1–5 years	**0.78**	**0.70**	**0.86**	**0.79**	**0.73**	**0.85**
	5+ years	0.95	0.83	1.08	**0.79**	**0.71**	**0.88**
	1–3 months (REF)	1.00			1.00		
Cancer subtype	Esophagus	**1.26**	**1.09**	**1.45**	**1.28**	**1.18**	**1.39**
	Gallbladder/Biliary tract	**1.15**	**1.02**	**1.30**	1.09	0.97	1.22
	Liver cancers	**1.41**	**1.17**	**1.71**	**1.34**	**1.21**	**1.48**
	Pancreas	**1.13**	**1.03**	**1.24**	1.07	0.99	1.16
	Small intestine	**1.39**	**1.07**	**1.81**	1.15	0.91	1.46
	Stomach	**1.19**	**1.05**	**1.35**	**1.15**	**1.06**	**1.26**
	Anal and colorectal (REF)	1.00			1.00		
Income quintile	1 (lowest)	1.11	1.00	1.24	**1.14**	**1.04**	**1.23**
	2	1.11	0.99	1.24	**1.10**	**1.01**	**1.20**
	3	1.09	0.97	1.22	**1.13**	**1.04**	**1.23**
	4	1.07	0.95	1.20	1.05	0.96	1.14
	5 (REF)	1.00			1.00		
Rural status	Rural	**2.06**	**1.88**	**2.26**	**1.81**	**1.69**	**1.94**
	Urban (REF)	1.00			1.00		
Year of death	2011–2014	1.02	0.93	1.11	1.05	0.98	1.12
	2015–2018	1.01	0.93	1.10	0.99	0.93	1.05
	2006–2010 (REF)	1.00			1.00		

Note significant results are bolded.

## Data Availability

All data used in the preparation of this paper is governed by privacy legislation from the province of Ontario and is not publicly available due to privacy and regulations governing the use of these data.

## Appendix A

**Table A1:** GI cancer cause of death codes.

Cancer Type	Cause of Death ICD-10 Code
Esophagus	C150, C151, C152, C153, C154, C155, C158, C159
Stomach	C160, C161, C162, C163, C165, C165, C166, C168, C169
Small Intestine	C170, C171, C172, C173, C178, C179
Colorectal	C180, C181, C182, C183, C184, C185, C186, C187, C188, C189, C19, C199, C200, C209
Anus and anal canal	C210, C211, C212, C218
Liver	C220, C222, C223, C224, C227, C228
Galbladder	C23, C239
Biliary tract	C221, C240, C241, C248, C249
Pancreas	C250, C251, C252, C253, C254, C257, C258, C259

**Table A2:** Data sources used.

Administrative Dataset	Data elements
Ontario Cancer Registry (OCR)	Cancer diagnosis and receipt of chemotherapy
Registered Persons Database (RPD)	Date of death, rural status, income quintile, postal code
Ontario Health Insurance Plan (OHIP)	Physician house call
Resident Assessment Instrument-Contact Assessment (RAICA)	Palliative home care nursing or support services
Homecare Database (HCD)	Palliative home care nursing or support services
National Ambulatory Care Reporting System (NACRS)	ED visits
Discharge Abstract Database (DAD)	Hospitalizations, Deyo–Charlson comorbidity index

## Appendix B

**Table A3:** Study population by year.

Year of Death	Study Population: Female	Study Population: Male
2006	1966	2663
2007	2070	2784
2008	2093	3011
2009	2183	2941
2010	2179	2845
2011	2319	3103
2012	2268	3145
2013	2354	3128
2014	2330	3210
2015	2370	3193
2016	2479	3448
2017	2392	3414
2018	2526	3569
